# Influence of Orthodontic-Related Complaints on Absenteeism and Presenteeism Among Japanese Workers

**DOI:** 10.7759/cureus.81384

**Published:** 2025-03-28

**Authors:** Yumi Nakano, Takashi Zaitsu, Ikuo Yonemitsu, Jun Aida, Takashi Ono

**Affiliations:** 1 Department of Orthodontic Science, Graduate School of Medical and Dental Sciences, Institute of Science Tokyo, Tokyo, JPN; 2 Department of Dental Public Health, Graduate School of Medical and Dental Sciences, Institute of Science Tokyo, Tokyo, JPN

**Keywords:** absenteeism, esthetics, occlusion, presenteeism, speech

## Abstract

Objective: Workplace health has become increasingly important in recent years. However, the cost of managing workers' health is enormous, and increasing labor productivity is important for improving it. The association between labor productivity and common oral diseases, such as dental caries, periodontal disease, and tooth loss, has been reported. The need for orthodontic care in the working-age population is currently increasing; however, no studies have examined the association between work performance and orthodontic-related complaints. Hence, this study examined the impact of orthodontic-related complaints on labor productivity among Japanese workers.

Materials and methods: The study involved 2,626 participants (2,136 males and 490 females; mean age: 44.8 years) who completed an internet-based self-reported survey. Participants were initially asked whether they had missed work, or been late, or left early due to oral health problems. For analysis, absenteeism (+) was defined as having at least one occurrence of (1) full-day absence, (2) half-day absence, or (3) late arrival/early departure, while absenteeism (-) was defined as having none. Presenteeism was assessed using the World Health Organization Health and Work Performance Questionnaire relative presenteeism score, with a threshold of 0.8 points. Logistic regression was used to examine associations between orthodontic-related complaints (esthetics, occlusion, speech) and absenteeism/presenteeism, with adjustment for sociodemographic and work factors.

Results: Workers with orthodontic-related complaints had a significantly higher risk of absenteeism and presenteeism compared with those without such complaints. Those worried about occlusion were 3.56 times more likely to experience absenteeism (95% CI, 2.12-6.00), while complaints about speech issues led to a 1.60 times higher risk of presenteeism (95% CI, 1.60-2.54).

Conclusions: This study quantitatively demonstrated the impact of orthodontic-related complaints on work productivity. This suggests the importance of incorporating orthodontic treatment into workplace health management programs and policy formulations to enhance labor productivity and reduce economic risks associated with absenteeism and presenteeism.

## Introduction

Worker health has become a priority recently. The World Health Organization (WHO) has emphasized the importance of health, safety, and welfare in the workplace, highlighting the mutual benefits for workers, companies, and countries [1]. Reforms are underway in Japan to achieve this goal [2]. However, the costs of worker healthcare are enormous, necessitating the enhancement of labor productivity to improve it. Absenteeism and presenteeism are indicators of labor productivity. Absenteeism refers to absence from work due to illness or accident. In contrast, presenteeism describes a situation where a worker is physically present in the workplace despite being incapacitated due to health problems [3,4]. The WHO Health and Work Performance Questionnaire (WHO/HPQ) is valuable for assessing labor productivity. This indicator is published and widely used by the WHO to quantitatively measure presenteeism, which is difficult to quantify [5]. Therefore, the financial losses associated with reduced labor efficiency have been calculated in various countries [6]. In Japan, the annual cost per worker includes US$1,165 for medical care, US$520 for absenteeism, and US$30,654 for presenteeism [7]. The cost of presenteeism is estimated at US$27 billion annually in Japan [8]. Effective health management is crucial to improving labor productivity and reducing costs for workers.

When considering health and labor productivity, oral diseases are currently a focus of attention. According to the WHO, productivity losses attributed to oral diseases are estimated to be approximately US$42 per person, with a global total of approximately US$323 billion [9]. Several studies have examined the association between oral diseases and work-related problems [10,11]. Although previous studies on the association between work and dental diseases have primarily focused on standard oral diseases, such as dental caries, periodontal disease, and tooth loss, studies that are not limited to workers have highlighted additional complaints related to esthetics, occlusion, and speech [12,13]. These issues are particularly strongly associated with orthodontic treatment. Additionally, orthodontic treatment includes a growing concern for esthetics among adults [14,15], which can have a significant psychosocial impact on patients [16]. Furthermore, the demand for orthodontic treatment in the working-age population is currently increasing [17-19].

Thus, orthodontic-related complaints, including esthetics, occlusion, and speech, are essential in the working-age population and significantly impact labor productivity. In particular, occlusion has been reported to affect physical exertion and concentration, making it more likely to influence workplace productivity. However, no studies have investigated the association between orthodontic-related complaints and labor performance, such as absenteeism and presenteeism, among those with orthodontic-related complaints. Therefore, this study aimed to investigate how the most common orthodontic-related complaints, esthetics, occlusion, and speech, are associated with worker productivity.

## Materials and methods

Data source

This cross-sectional study used data obtained from an Internet-based self-reported questionnaire survey conducted in February 2016. Participants were initially recruited through services managed by Macromill, Inc. Upon registration, participants provided written consent. The recruited aimed to cover all occupations and gather a total of 3,000 people from all 11 occupational categories of the Ministry of Health, Labor, and Welfare, with equal gender representation. Macromill, Inc. boasts the no. 1 track record in Japan in online research, and although the target audience for their monitors is limited to those aged ≥20 years, it encompasses a wide range of subjects. Two thousand six hundred and twenty-six (2,136 males and 490 females) workers (mean age: 44.8 ± 10.2 years) were recruited within the recruitment deadline. The inclusion criterion was workers aged 20 years or older. This study was approved and conducted with the approval of the Ethics Review Board of the Faculty of Dentistry, Tokyo Medical and Dental University (approval number: D2015-526).

Dependent variable: work performance

Absenteeism

The number of absences due to oral health problems was considered a dependent variable for absenteeism. Participants were asked the following question: “In the past year, have you missed work or been late or left early for oral health problems? ” In response, participants were asked to answer the following questions: (1) the number of days they missed work, (2) the number of days they took off for half a day, and (3) the number of days they came to work late or left work early. In the analysis, absenteeism (+) was defined as having at least one occurrence of (1), (2), or (3), and absenteeism was defined (-) as having no occurrences.

Presenteeism

Presenteeism was assessed using the WHO-HPQ [5,20]. We utilized the validated Japanese version of the HPQ short form, which was translated into Japanese and independently back-translated to English [21]. The WHO-HPQ presenteeism scores include two forms: absolute and relative presenteeism. Absolute presenteeism was calculated as the difference between an individual’s score over the past 28 days and the score for an average worker in the same job. Relative presenteeism score was computed as the ratio of an individual’s score to others' scores [20]. Given that the Japanese tend to have low self-evaluations [22], relative presenteeism was used as the evaluation item [23]. A relative presenteeism score of ≥0.8 was categorized as presenteeism (+), while a score of <0.8 was categorized as presenteeism (-) [24].

Independent variables: orthodontic-related complaints

Orthodontic-related complaints were considered independent variables. Participants were asked the following question: “Do you have any complaints about the condition of your teeth or mouth? Answer with yes or no. Among the options for this question, the following were identified as orthodontic-related complaints: (1) esthetics, (2) occlusion, and (3) speech.

Covariates

Sociodemographic information and worker characteristics strongly associated with complaints about oral problems, such as occupational classification and work shift, were included as covariates. Sociodemographic information included age (20-29, 30-39, 40-49, 50-59, and 60 years or older), sex, family income (<¥2 million, ¥2-3.9 million, ¥4-5.9 million, ¥6-7.9 million, ¥8-9.9 million, ¥10-11.9 million, ¥12-14.9 million, ¥15-19.9 million, ≧¥20 million, and unknown), smoking status, alcohol consumption, diabetes, stroke, heart disease, periodontal disease, and the number of own teeth. Workers' occupations were classified into 11 categories based on the Japanese Standard Occupational Classification, each corresponding to a specific job category (Table 1) [25,26]. Work shifts were classified into day shifts, night shifts, day and night shifts, flexes, and other work shifts.

**Table 1:** Japanese Standard Occupational Classification [25]

Occupation classification	Example job titles	Category
(a) Administrative professionals	Company officers, company administrative officers, and public administrative officers	White collar
(b) Professional and technical professionals	Research, health professionals, faculty	
(c) Office workers	Human affairs, labor, accounting, and management	
(d) Marketing personnel	Sales	
(e) Service professionals	Facilities and equipment management, custom centers, home helpers, beauty technicians, etc.	
(f) Security occupational workers	Self-defense officers, police officers, security officers	Blue collar
(g) Agricultural, forestry, and fishery workers	Landscapers, fishermen, agricultural officers	
(h) Production process workers	Steel maintenance control and monitoring workers, gum and plastic product manufacturing workers	
(i) Transportation and machinery drivers	Taxi drivers, bus drivers, and machinery operators	
(j) Construction and mining workers	Carpenters, plumbers, civil engineers	
(k) Personnel involved in transportation, cleaning, and packaging	Delivery personnel, cleaning personnel	

Statistical analysis

The descriptive distribution of orthodontic-related complaints was analyzed based on age, sex, occupational classification, family income, work shifts, smoking habits, drinking habits, presence of systemic disease, and number of teeth. A survey was conducted to determine the percentages of absenteeism and presenteeism due to orthodontic-related complaints.

Logistic regression analysis was performed with absenteeism and presenteeism as the dependent variables and orthodontic-related complaints (esthetics, occlusion, and speech) as the independent variables. Multiple models were constructed to examine the mediating effects of sociodemographic and worker characteristics as mediators. First, univariate analysis was performed (Model 1). At this stage, univariate analysis was conducted separately for each of the three types of orthodontic-related complaints. Second, all covariates were included in the model for each dependent variable (Model 2). In this model, all covariates were included for each of the three types of orthodontic-related complaints. Finally, all independent variables and covariates were then included in the model (Model 3). This model included all three types of orthodontic-related complaints and all covariates.

Statistical analysis was performed using SPSS Statistics version 28 (IBM Corp., Released 2021. IBM SPSS Statistics for Windows, Version 28.0. Armonk, NY: IBM Corp). The significance level was set at 5%, with a 95% confidence interval (CI). In this study, we used ordinary variance estimation instead of robust analysis because the sample size was large and no outliers were observed. We also confirmed that the two methods yielded similar results.

## Results

Table 2 shows the descriptive distribution of orthodontic-related complaints according to age, sex, occupational classification, family income, work shift, smoking habits, drinking habits, presence of systemic disease, and number of teeth. The participants in this study were 2,626 workers (2,136 males and 490 females). The mean age of the participants was 44.8 ± 10.2 years. Among the participants, 612 (23.3%), 559 (21.3%), and 233 (8.9%) reported complaints about esthetics, occlusion, and speech, respectively.

**Table 2:** Descriptive distribution of oral concerns by age, sex, occupational classification, family income, work shifts, smoking, drinking, the presence of systemic disease, and the number of teeth

		Total	Esthetics		Occlusion		Speech	
		N=2626	Complaints (N=612)	No complaints (N=2,014)	Complaints (N=559)	No complaints (N=2,067)	Complaints (N=233)	No complaints (N=2,393)
		N(%)/mean	％/mean	％/mean	％/mean	％/mean	％/mean	％/mean
Age (years)	20s	226 (8.6%)	10.8%	7.9%	7.5%	8.9%	3.9%	10.0%
	30s	599 (22.8%)	24.3%	22.3%	19.7%	23.7%	8.7%	27.1%
	40s	869 (33.1%)	34.8%	32.6%	33.3%	33.0%	11.4%	39.7%
	50s	742 (28.3%)	24.0%	29.5%	30.2%	27.7%	9.8%	33.9%
	60 years or older	190 (7.2%)	6.0%	7.6%	9.3%	6.7%	4.2%	8.1%
Sex	Male	2,136 (81.3%)	76.0%	83.0%	80.9%	81.5%	31.2%	96.6%
	Female	490 (18.7%)	24.0%	17.0%	19.1%	18.5%	6.9%	22.2%
Occupational	White	787 (30.0%)	22.7%	32.2%	28.6%	30.3%	10.3%	35.9%
Classification	Blue	1,839 (70.0%)	77.3%	67.8%	71.4%	69.7%	27.8%	82.9%
Family income	<2 million Yen (reference)	87 (3.3%)	2.9%	3.4%	4.7%	3.0%	1.0%	4.0%
	2–3.9 million Yen	583 (22.2%)	25.0%	21.4%	22.2%	22.2%	9.5%	26.1%
	4–5.9 million Yen	725 (27.6%)	28.3%	27.4%	30.1%	26.9%	13.6%	31.9%
	6–7.9 million Yen	458 (17.4%)	15.7%	18.0%	15.9%	17.9%	4.1%	21.5%
	8–9.9 million Yen	267 (10.2%)	10.9%	9.9%	9.5%	10.4%	4.6%	11.9%
	10–11.9 million Yen	141 (5.4%)	3.9%	5.8%	4.5%	5.6%	2.0%	6.4%
	12–14.9 million Yen	104 (4.0%)	2.9%	4.3%	3.6%	4.1%	0.7%	5.0%
	15–20 million Yen	48 (1.8%)	1.6%	1.9%	1.3%	2.0%	0.3%	2.3%
	≧20 million Yen	46 (1.8%)	1.0%	2.0%	2.3%	1.6%	0.7%	2.1%
	Unknown/blank	167 (6.4%)	7.7%	6.0%	6.1%	6.4%	1.8%	7.7%
Work shifts	Day shift	2,001 (76.2%)	72.9%	77.2%	73.2%	77.0%	28.1%	90.8%
	Both day and night shifts	59 (2.2%)	3.8%	1.8%	4.1%	1.7%	1.6%	2.4%
	Night shift	437 (16.6%)	18.0%	16.2%	17.9%	16.3%	6.9%	19.6%
	Flex	84 (3.2%)	2.8%	3.3%	3.0%	3.2%	1.0%	3.9%
	Other	45 (1.7%)	2.6%	1.4%	1.8%	1.7%	0.5%	2.1%
Smoking	Current	789 (30.0%)	34.3%	28.7%	34.5%	28.8%	13.7%	35.0%
	Past	613 (23.3%)	24.0%	23.1%	26.3%	22.5%	10.6%	27.2%
	No	1,224 (46.6%)	41.7%	48.1%	39.2%	48.6%	13.7%	56.6%
Drinking	Everyday	757 (28.8%)	32.0%	27.9%	33.8%	27.5%	13.2%	33.6%
	Sometimes	910 (34.7%)	33.2%	35.1%	33.3%	35.0%	12.4%	41.4%
	No	959 (36.5%)	34.8%	37.0%	32.9%	37.5%	12.4%	43.8%
Disease	Diabetes	137 (5.2%)	6.9%	4.7%	7.9%	4.5%	3.9%	5.6%
	Stroke	17 (0.6%)	2.0%	0.2%	2.0%	0.3%	1.8%	0.3%
	Heart disease	46 (1.8%)	2.8%	1.4%	4.7%	1.0%	2.9%	1.4%
	Periodontal disease	34 (1.3%)	2.6%	0.9%	2.9%	0.9%	1.3%	1.3%
Number of teeth		24.62 ± 6.05	23.520	24.95	23.06	25.04	21.46	24.935

Table 3 presents the association between orthodontic-related complaints and workplace performance. The percentage of participants who were absent from work due to oral health problems was 3.7%, and 9.6% had presenteeism. Regarding all complaints (esthetics, occlusion, and speech), those with problems were significantly more likely to have problems with absenteeism than presenteeism.

**Table 3:** Descriptive association between oral concerns and work performance WHO/HPQ: World Health Organization Health and Work Performance Questionnaire

		Total		Esthetics				Occlusion				Speech			
		(N=2626)		(+) (N=612)		(−) (N=2014)		(+) (N=559)		(−) (N=2,067)		(+) (N=233)		(−) (N=2,393)	
		N	％	N	％	N	％	N	％	N	％	N	％	N	％
Absenteeism (absent due to oral problems)															
(+)		97	3.7	43	7.0	54	2.7	53	9.5	44	2.1	26	11.2	71	3.0
(−)		2529	96.3	569	93.0	1960	97.3	506	90.5	2023	97.9	207	88.8	2322	97.0
Presenteeism (WHO/HPQ)															
(+)		252	9.6	79	12.9	173	8.6	66	11.8	186	9.0	36	15.5	216	9.0
(−)		2374	90.4	533	87.1	1841	91.4	493	88.2	1881	91.0	197	84.5	2177	91.0

Table 4 shows the results of a multivariable logistic regression analysis of the association between absenteeism and worry. Univariate logistic regression analysis in Model 1 showed that all complaints were significantly associated with higher absenteeism (E: OR=2.74, 95% CI 1.81-4.13, O: OR=4.82, 95% CI 3.19-7.27, S: OR=4.11, 95% CI 2.56-6.58). The observed prevalence of absenteeism was higher in occlusion and speech anxiety than in esthetic anxiety. After adjusting for all covariates, logistic regression analysis on each independent variable showed the same conclusions in Model 2 (E: OR=2.53 95% CI 1.62-3.94, O: OR=4.41 95% CI 2.85-6.85, S: OR=3.61 95% CI 2.12-6.14) and occlusal anxiety was significantly associated with absenteeism in Model 3 (OR=3.56, 95% CI 2.12-6.00).

**Table 4:** Logistic regression analysis of the association between esthetics, occlusion, speech, and absenteeism Model 1: univariate analysis. Model 2: multivariable analysis adjusted for all covariates for each dependent variable. Model 3: multivariable analysis adjusted for all independent variables and covariates. CI: confidence interval, OR: odds ratio

		Model 1				Model 2				Model 3			
			95％ CI				95％ CI				95％ CI		
		OR	Lower	Upper	p	OR	Lower	Upper	p	OR	Lower	Upper	p
Esthetics	（-）(reference)												
	（＋）	2.743	1.818	4.139	<0.001	2.528	1.622	3.939	<0.001	1.116	0.65	1.916	0.691
Occlusion	（-）(reference)												
	（＋）	4.816	3.191	7.267	<0.001	4.414	2.845	6.848	<0.001	3.561	2.116	5.991	<0.001
Speech	（-）(reference)												
	（＋）	4.108	2.564	6.581	<0.001	3.609	2.121	6.141	<0.001	1.806	0.995	3.279	0.052
Age (years)	20s (reference)	-	-	-	-	-	-	-	-				
	30s	-	-	-	-	-	-	-	-	1.018	0.427	2.43	0.967
	40s	-	-	-	-	-	-	-	-	0.825	0.348	1.954	0.662
	50s	-	-	-	-	-	-	-	-	0.636	0.26	1.559	0.323
	60 years or older	-	-	-	-	-	-	-	-	0.438	0.136	1.409	0.166
Sex	Male (reference)	-	-	-	-	-	-	-	-				
	Female	-	-	-	-	-	-	-	-	1.126	0.613	2.069	0.702
Occupational classification	White (reference)	-	-	-	-	-	-	-	-				
	Blue	-	-	-	-	-	-	-	-	0.802	0.487	1.319	0.385
Family income	<2 million Yen (reference)	-	-	-	-	-	-	-	-				
	2–3.9 million Yen	-	-	-	-	-	-	-	-	0.29	0.094	0.89	0.031
	4–5.9 million Yen	-	-	-	-	-	-	-	-	0.552	0.197	1.544	0.257
	6–7.9 million Yen	-	-	-	-	-	-	-	-	0.643	0.221	1.868	0.417
	8–9.9 million Yen	-	-	-	-	-	-	-	-	0.618	0.196	1.955	0.413
	10–11.9 million Yen	-	-	-	-	-	-	-	-	0.471	0.115	1.937	0.297
	12–14.9 million Yen	-	-	-	-	-	-	-	-	1.183	0.337	4.146	0.793
	15–20 million Yen	-	-	-	-	-	-	-	-	2.088	0.521	8.364	0.299
	≧20 million Yen	-	-	-	-	-	-	-	-	1.939	0.484	7.774	0.350
	Unknown/blank	-	-	-	-	-	-	-	-	0.305	0.069	1.355	0.119
Work shifts	Day shift (reference)	-	-	-	-	-	-	-	-				
	Night shift	-	-	-	-	-	-	-	-	0.285	0.037	2.201	0.229
	Both day and night shifts	-	-	-	-	-	-	-	-	0.46	0.221	0.956	0.038
	Flex	-	-	-	-	-	-	-	-	0.98	0.334	2.879	0.971
	Others	-	-	-	-	-	-	-	-	0	0	-	0.997
Smoking	No (reference)	-	-	-	-	-	-	-	-				
	Past	-	-	-	-	-	-	-	-	1.543	0.901	2.645	0.114
	Current	-	-	-	-	-	-	-	-	1.353	0.741	2.471	0.326
Drinking	No (reference)	-	-	-	-	-	-	-	-				
	Sometimes	-	-	-	-	-	-	-	-	1.447	0.822	2.548	0.200
	Everyday	-	-	-	-	-	-	-	-	1.099	0.628	1.922	0.740
Diabetes	None	-	-	-	-	-	-	-	-				
	Present	-	-	-	-	-	-	-	-	1.794	0.781	4.118	0.168
Stroke	None	-	-	-	-	-	-	-	-				
	Present	-	-	-	-	-	-	-	-	1.262	0.225	7.084	0.791
Heart disease	None	-	-	-	-	-	-	-	-				
	Present	-	-	-	-	-	-	-	-	0.644	0.155	2.672	0.544
Periodontal disease	None	-	-	-	-	-	-	-	-				
	Present	-	-	-	-	-	-	-	-	5.253	2.004	13.767	<0.001
Number of teeth		-	-	-	-	-	-	-	-	0.984	0.952	1.018	0.353

Table 5 shows the results of a multivariable logistic regression analysis of the association between presenteeism and complaints. Univariate logistic regression analysis revealed that all complaints were significantly associated with presenteeism (E: OR=1.58, 95% CI 1.19-2.10, O: OR=1.35, 95% CI 1.01-1.82, S: OR=1.84, 95% CI 1.26-2.70). The observed prevalence of presenteeism was higher for speech and esthetic anxiety than for occlusion anxiety. After adjusting for all covariates, logistic regression analysis on each independent variable showed the same conclusions in Model 2 (E: OR=1.50 95% CI 1.12-2.01, O: OR=1.38 95% CI 1.02-1.89, S: OR=1.91 95% CI 1.27-2.85) and speech anxiety was significantly associated with presenteeism in Model 3 (OR=1.60 95% CI 1.60-2.54).

**Table 5:** Logistic regression analysis of the association between esthetics, occlusion, speech, and presenteeism Model 1: univariate analysis. Model 2: multivariable analysis adjusted for all covariates for each dependent variable. Model 3: multivariable analysis adjusted for all independent variables and covariates. OR: odds ratio, CI: confidence interval

		Model 1				Model 2				Model 3			
			95％ CI				95％ CI				95％ CI		
		OR	Lower	Upper	p	OR	Lower	Upper	p	OR	Lower	Upper	p
Esthetics	（-）(reference)												
	（＋）	1.577	1.188	2.093	0.002	1.497	1.115	2.011	0.007	1.28	0.903	1.815	0.166
Occlusion	（-）(reference)												
	（＋）	1.354	1.005	1.824	0.046	1.384	1.015	1.887	0.040	1.069	0.739	1.547	0.723
Speech	（-）(reference)												
	（＋）	1.842	1.257	2.698	0.002	1.905	1.271	2.854	0.002	1.601	1.011	2.536	0.045
Age (years)	20s (reference)	-	-	-	-	-	-	-	-				
	30s	-	-	-	-	-	-	-	-	0.795	0.507	1.246	0.317
	40s	-	-	-	-	-	-	-	-	0.618	0.391	0.976	0.039
	50s	-	-	-	-	-	-	-	-	0.533	0.325	0.873	0.012
	60 years or older	-	-	-	-	-	-	-	-	0.574	0.285	1.154	0.119
Sex	Male (reference)	-	-	-	-	-	-	-	-				
	Female	-	-	-	-	-	-	-	-	1.507	1.076	2.109	0.017
Occupational classification	White (reference)	-	-	-	-	-	-	-	-				
	Blue	-	-	-	-	-	-	-	-	0.872	0.636	1.197	0.397
Family income	<2 million Yen (reference)	-	-	-	-	-	-	-	-				
	2–3.9 million Yen	-	-	-	-	-	-	-	-	0.625	0.328	1.191	0.153
	4–5.9 million Yen	-	-	-	-	-	-	-	-	0.538	0.281	1.027	0.060
	6–7.9 million Yen	-	-	-	-	-	-	-	-	0.583	0.297	1.143	0.116
	8–9.9 million Yen	-	-	-	-	-	-	-	-	0.391	0.180	0.848	0.017
	10–11.9 million Yen	-	-	-	-	-	-	-	-	0.663	0.294	1.492	0.321
	12–14.9 million Yen	-	-	-	-	-	-	-	-	0.165	0.045	0.605	0.007
	15–20 million Yen	-	-	-	-	-	-	-	-	0.380	0.100	1.439	0.154
	≧20 million Yen	-	-	-	-	-	-	-	-	0.273	0.057	1.296	0.102
	Unknown/blank	-	-	-	-	-	-	-	-	0.648	0.305	1.374	0.258
Work shifts	Day shift (reference)	-	-	-	-	-	-	-	-				
	Night shift	-	-	-	-	-	-	-	-	0.163	0.022	1.192	0.074
	Both day and night shifts	-	-	-	-	-	-	-	-	1.104	0.775	1.572	0.585
	Flex	-	-	-	-	-	-	-	-	0.804	0.340	1.898	0.618
	Others	-	-	-	-	-	-	-	-	1.424	0.586	3.460	0.435
Smoking	No (reference)	-	-	-	-	-	-	-	-				
	Past	-	-	-	-	-	-	-	-	1.265	0.909	1.759	0.163
	Current	-	-	-	-	-	-	-	-	1.236	0.862	1.771	0.249
Drinking	No (reference)	-	-	-	-	-	-	-	-				
	Sometimes	-	-	-	-	-	-	-	-	0.888	0.621	1.270	0.516
	Everyday	-	-	-	-	-	-	-	-	0.924	0.677	1.262	0.620
Diabetes	None	-	-	-	-	-	-	-	-				
	Present	-	-	-	-	-	-	-	-	0.786	0.379	1.629	0.517
Stroke	None	-	-	-	-	-	-	-	-				
	Present	-	-	-	-	-	-	-	-	0.917	0.171	4.912	0.919
Heart disease	None	-	-	-	-	-	-	-	-				
	Present	-	-	-	-	-	-	-	-	0.962	0.302	3.071	0.948
Periodontal disease	None	-	-	-	-	-	-	-	-				
	Present	-	-	-	-	-	-	-	-	2.698	1.101	6.611	0.030
Number of teeth		-	-	-	-	-	-	-	-	1.013	0.989	1.038	0.292

## Discussion

The results of this study suggest that orthodontic-related complaints are associated with absenteeism and presenteeism, which is not limited to dentistry, even after adjusting for socioeconomic status. Among the three orthodontic-related complaints, occlusion was strongly associated with absenteeism, and speech was strongly associated with presenteeism. Moreover, the findings of this study align with previous research, which has shown that oral problems are associated with high absenteeism. In prior studies, periodontal disease has been related to presenteeism among workers. The participants in this study were Japanese workers surveyed via the Internet, and their characteristics were similar and comparable to those reported in previous studies on absenteeism and the WHO-PHQ rates conducted in Japan [11,24]. In contrast to previous studies, this study is novel in that it examined orthodontic-related complaints.

Although orthodontic-related complaints are associated with absenteeism and presenteeism, occlusion is especially associated with absenteeism, and the potential reasons are discussed below. Generally, factors that lead to a poor bite include dental caries, tooth loss, and poor dental alignment (including plexus, open bite, and mandibular prognathism). However, since the number of teeth was adjusted in this study, it suggests that dental caries or misaligned teeth, rather than tooth loss, may be associated with absenteeism. Additionally, malocclusion has been reported to affect the body's overall condition, causing indigestion [27-29]. Hence, these factors may be associated with absenteeism. It is also possible that severe dental caries leading to bite problems were directly associated with absenteeism; however, this study did not investigate the severity of dental caries or indigestion. Further research should be conducted to investigate these factors.

On the other hand, speech was especially associated with presenteeism. Speech problems can significantly impact communication and customer service in the workplace. These issues may be caused by the alignment of teeth that require orthodontic treatment, such as mandibular prognathism or an open bite [30]. Although no studies have been conducted to determine whether the workers in this study were employed in customer service-related jobs, it is necessary to investigate the effects of speech-related presenteeism across various occupational roles.

The participants in this study were Japanese workers. According to the latest Japanese Health Statistics Survey, 7.7% of the Japanese population is undergoing orthodontic treatment [14]. Since the number of men tends to be low, introducing orthodontic treatment as an approach to improve the labor productivity of Japanese workers may be necessary in the future. The number of individuals undergoing orthodontic treatment may increase in the future, particularly as mask-wearing during the COVID-19 pandemic may encourage more people to pursue orthodontic care. However, detailed examination parameters for determining the necessity of orthodontic treatment have not yet been incorporated into dental examinations in Japan. It is important to understand the need for future orthodontic treatment by conducting dental examinations focusing on it.

A key strength of this study is that it is the first study to investigate the association between labor performance (absenteeism and presenteeism) and orthodontic-related complaints, along with the relationship between WHO/HPQ scores (overall presenteeism) and dental problems in absenteeism. However, a limitation of this study is its cross-sectional design, which does not allow for establishing a causal relationship. Therefore, it is necessary to conduct a follow-up, rather than a cross-sectional study, to investigate this association. Furthermore, the investigation of confounding factors leading to the present results may have been insufficient. Specifically, psychological stress and similar factors should be investigated, and mediation analysis may also be necessary. Additionally, since we do not have accurate clinical data on orthodontic treatment and whether patients received orthodontic treatment, we cannot ascertain whether the problems were caused by issues that could only be addressed by orthodontic treatment. Specifically, non-differential misclassification, where inaccuracies occur uniformly across different groups, could introduce a bias towards the null. Additionally, this study depends on staff scheduling and flexibility among countries. Moreover, this study did not distinguish between planned absenteeism and unexpected absenteeism. In the future, it may be necessary to investigate the association between labor performance and the presence or absence of orthodontic treatment, detailed clinical information on orthodontic treatment, information on health status, and whether absences or early departures were planned.

## Conclusions

Providing workers with orthodontic treatment to address all these issues is essential; in particular, approaches to improving occlusion and speech are important for work performance. In addition, this study used data from 2016, and people may be becoming increasingly aware of orthodontic treatments because of the COVID-19 pandemic. A more detailed survey of orthodontic treatments is necessary to confirm these findings.
